# Push Button Population Health: The SMART/HL7 FHIR Bulk Data Access Application Programming Interface

**DOI:** 10.1038/s41746-020-00358-4

**Published:** 2020-11-19

**Authors:** Kenneth D. Mandl, Daniel Gottlieb, Joshua C. Mandel, Vladimir Ignatov, Raheel Sayeed, Grahame Grieve, James Jones, Alyssa Ellis, Adam Culbertson

**Affiliations:** 1 Computational Health Informatics Program, Boston Children’s Hospital, Boston, MA USA; 2 Departments of Pediatrics, Harvard Medical School, Boston, MA USA; 3 Departments of Biomedical Informatics, Harvard Medical School, Boston, MA USA; 4 Central Square Solutions, Cambridge, MA USA; 5 Microsoft Healthcare, Redmond, WA USA; 6 Health Level 7, Ann Arbor, MI USA; 7 Health Intersections, Pty Ltd, Warrandyte, Australia

**Keywords:** Health policy, Outcomes research

## Abstract

The 21st Century Cures Act requires that certified health information technology have an application programming interface (API) giving access to all data elements of a patient’s electronic health record, “without special effort”. In the spring of 2020, the Office of the National Coordinator of Health Information Technology (ONC) published a rule—21st Century Cures Act Interoperability, Information Blocking, and the ONC Health IT Certification Program—regulating the API requirement along with protections against information blocking. The rule specifies the SMART/HL7 FHIR Bulk Data Access API, which enables access to patient-level data across a patient population, supporting myriad use cases across healthcare, research, and public health ecosystems. The API enables “push button population health” in that core data elements can readily and standardly be extracted from electronic health records, enabling local, regional, and national-scale data-driven innovation.

## Introduction

A new Department of Health and Human Services rule^1^, implementing health information technology provisions of the 21st Century Cures Act, has potential to greatly accelerate the use of population data at health-system scale. In a healthcare system managing an aging population with a diminishing primary care workforce, innovation in wellness, treatments, care delivery, and public health requires ready access to data on populations.

A fundamental barrier is that the largest source of detailed clinical information—the electronic health record (EHR), is quite difficult to access. Extracting data from EHRs requires a heavy lift—one that is often beyond capabilities of healthcare practices and even many major medical centers. Another is that payor claims data on populations has not been readily available to providers in a turnkey fashion. Hence, the transformation of medicine to a data-driven enterprise has been slow and fraught.

A learning health system^2^, requires a transition from reliance on traditional “evidence-based medicine”, where the patient is assessed in the context of the published clinical trials literature, to what is sometimes playfully referred to as “medicine-based evidence”^3^, putting the patient in the context of data. For example, a patient may be compared to a population of similar patients to understand risks, trajectories, and outcomes^4,5^. Or machine-learning algorithms, hungry for large datasets, may be tuned to drive individual care, based on population-derived intelligence.

For cost-efficiency and improved outcomes, the value of care delivered must be easily measurable and provider organizations held accountable for their performance^6^. Computable electronic clinical quality measures to assess healthcare quality based on structured data collected during the process of patient care are not readily produced by today’s health information technology^7^.

Public health’s limited access to intelligence based on clinical data resources has been thrown into extraordinary relief in the midst of the COVID-19 pandemic. In March 2020, the Centers for Disease Control and Prevention (CDC) only had clinical data on underlying health conditions for 5.8% of COVID-19 cases^8^. There is a dire need for real-time clinical data to power robust biosurveillance.

There are myriad additional uses of easily and uniformly produced population health data, including post-marketing surveillance of therapeutics and devices, building datasets to develop and tune machine-learning algorithms, and comparative effectiveness research.

Today, many organizations lack expertise to efficiently use EHR data for analytics. Because EHR systems are not themselves nimble analytic or data sharing platforms, larger healthcare organizations often manage patient populations by extracting, transforming, and loading (ETL) EHR data into an analytic platform. This often requires a team of IT professionals to develop and maintain mappings between the source and target data schemas and to maintain the infrastructure that handles the export and import operations. These manual processes are expensive and time-consuming and require expertise that may not be locally available. Certainly, this capacity would be well-beyond most provider practices, denying them the benefits of the data that they enter at great burden^9^.

### Lack of uniform data standardization

Another important issue is that even when data are extracted from EHRs, the formats are non-standard and therefore difficult to combine and analyze. This is further complicated when data exported from EHRs is linked with data derived from healthcare claims which are often made available by payor organizations in non-standard formats as well. Large scientific and healthcare communities have harmonized and shared data over i2b2, PCORNet, and Observational Medical Outcomes Partnership (OMOP)/Observational Health Data Sciences and Informatics (OHDSI) ontologies. Extensive tooling around these standards has been developed to make data useful for analysis, but owing to the level of custom, site-specific data mapping required, community participation tends to be limited to large academic medical centers with specialized, and often grant-funded teams.

Further, the paradigm of data sharing between a healthcare organization and numerous entities—from public health authorities, to payors, to accreditors—depends on producing periodic static reports^1^. A hospital IT department may need to produce hundreds of reports that incorporate essentially the same data elements in different formats and on different schedules for multiple stakeholders.

The 21st Century Cures Act, signed in 2016, requires that certified health information technology have an application programming interface (API) giving access to all data elements of a patient’s EHR, “without special effort”. In the spring of 2020, the Office of the National Coordinator of Health Information Technology (ONC) published a rule—21st Century Cures Act Interoperability, Information Blocking, and the ONC Health IT Certification Program^1^—regulating the API requirement along with protections against information blocking. The rule covers two open APIs for obtaining data in the Health Level Seven Fast Healthcare Interoperability Resources (HL7^®^ FHIR^®^) standard. Both of these APIs were inspired by successful examples in industry, including Apple’s early success in using the App Store API to cleanly separate third-party app development from the smartphone platform^10^ and the widespread industry adoption of open standards such as representational state transfer (RESTful) APIs for data transfer and OAuth authorization.

The SMART on FHIR API primarily provides access to data on individual patients and supports use cases for both healthcare providers and patients. SMART on FHIR, funded by ONC, enables apps to read or write data from EHRs essentially turning EHRs into smartphone-like app platforms. Two striking examples that leverage this standard now are: (1) Apple’s Health app^11^ that connects across the SMART API to hundreds of healthcare institutions, enabling patients to have a consolidated view of their healthcare data across multiple sites of care; and (2) the Centers for Medicare and Medicaid (CMS) Blue Button 2.0 API that uses SMART on FHIR to provision historical claims data to beneficiaries, supporting apps with capabilities like making plan recommendations based on healthcare utilization (https://bluebutton.cms.gov/).

The second API, SMART/HL7 FHIR Bulk Data Access—the focus of this manuscript—enables access to patient-level data across a patient population, supporting the many use cases described above. We build on the success of SMART on FHIR, 8 years after the original SMART project under ONC’s Strategic Health IT Advanced Research Projects (SHARP) Program began. ONC catalyzed the next step, asking the SMART Health IT Team and HL7 to define a second API. It would handle data on cohorts with multiple patients rather than just one patient at a time. The result is a now-regulated capacity, required in certified health information technology by 2022, to export FHIR data, from any EHR, in an easily consumable NDJSON formatted flat file.

Mandl and Kohane’s article in Nature Biotechnology, Federalist Principles for Federated Healthcare Networks^12^, explored instrumenting the health system to share and use data at scale, across sites of care. It raised the question of how to put software into health systems that is maintained beyond the life of a particular grant. The article suggested that if there is a federated network, health systems need to be engaged as fully participatory members. But getting health systems to participate is challenging. When National Coordinator of Health Information Technology, Dr. Donald Rucker read this article, he observed, “If the health system is going to care about data, those data should be about payment”. Rucker suggested examining SMART on FHIR as a model for exporting data in a principled, reproducible way, with an initial focus on information exchange between payors and providers. This discussion led to an initial meeting in December 2017 to discuss the FHIR bulk data API proposal, identify and prioritize use cases, and consider pathways to regulation^13^. Following that meeting—attended by ONC leadership, HL7, the SMART Team, public and private payors, large technology companies, and health-system representatives—the Office of the National Coordinator of Health Information technology funded the SMART team to work with HL7 on design of an API analogous to the SMART on FHIR, but for population-level data inquiry. We describe here, the development of the standard, the tools to support it, and the uptake in the community.

## Results

### SMART/HL7 FHIR Bulk Data Access API

The SMART/HL7 FHIR Bulk Data Access API has been rapidly defined, standardized, and piloted. With efforts by the SMART Health IT team, ONC, CMS, HL7, big cloud vendors and the FHIR community, including the Argonaut FHIR accelerator, the API allows the ready extraction of standardized health information from EHRs at system scale. It builds on the success of the SMART on FHIR API, which standardized a method for connecting and authorizing an app to an EHR. By 2022, certified health information technology will require a bulk FHIR server whereby the organization that owns the IT (e.g., EHR or cloud-hosted FHIR server) can allow authorized software clients to interrogate the server and return population datasets. To limit impact of bulk data queries on EHR performance, the specification enables asynchronous coarse-grained queries against the API. There is an expectation that further manipulation and computation will be performed in well-suited analytic environments. Versions of it, described below, have been adapted by CMS to provision-claim data to providers.

SMART/HL7 Bulk Data will provide, from all certified health IT, data in a uniform, standardized, computable format. Hence analytic processes (R code or SAS routines) can be created once and run anywhere in the healthcare system, directly on the bulk data export files, or the files can be loaded into open or proprietary analytic environments for improved computational efficiency^14^. This standardization permits analytics at scale. There are also increasing transforms available between standards (e.g., FHIR to i2b2, OMOP to FHIR), enabling bulk FHIR exports to have a natural fit in a larger ecosystem. In addition, a SMART/HL7 bulk export apps model is possible, similar to the SMART on FHIR apps model. A bulk FHIR apps section has been added to the SMART App Gallery (https://apps.smarthealthit.org/apps/category/bulk-data).

Because the ONC rule implementing the 21st Century Cures Act IT provisions will be in full force by 2022, we can expect that “push button” population health capacity^13^ afforded by the API will be universally available. Shareable, standardized data on populations can be analyzed using the same systems and routines across the healthcare system. The data that will be made available via the API are defined by the U.S. Core Dataset for Interoperability (USCDI), which will be augmented over time (https://www.healthit.gov/isa/united-states-core-data-interoperability-uscdi).

### API design

Designed to handle large datasets, the SMART/HL7 FHIR Bulk Data Access API is an asynchronous request framework with multiple operations controlled via parameters that leverage a standardized system-to-system authorization framework—SMART Backend Services Authentication and Authorization^13^.

#### Kickoff request

The specification covers the following HTTP kickoff requests to one of the server’s exposed export operation endpoints using a required ‘Prefer: respond-async’ HTTP header:

- [FHIR Server Base]/Patient/$export—export data on all patients
- [FHIR Server Base]/Group/[group id]/$export—export all data for patients within a specified group (e.g., accountable care cohort, research group, health plan members)
- [FHIR Server Base]/$export—full system-level export of all resources

Data are returned as a series of files, each containing one type of FHIR resource. While all bulk data servers must have the ability to return data in the widely adopted Newline Delimited JSON (NDJSON) format, servers may also optionally be able to return the data in other file formats. Query parameters have been defined to allow applications to request output in other formats and/or filter the result set, as described in Table 1. Servers may also further limit data returned based on policies and regulations.

**Table 1:** Key query parameters to fine-tune bulk FHIR files generated from the kickoff request.

Parameter (string)	Context
_*outputFormat*	Client applications can optionally request files in a format other than NDJSON if the server supports it.
*_since*	Filter results to only FHIR resources updated after the given timestamp.
*_type*	Filter results to those of the specified FHIR resource types.
_*typeFilter*	Experimental syntax to express complex filter conditions.

#### Status requests

**Fig. 1:**
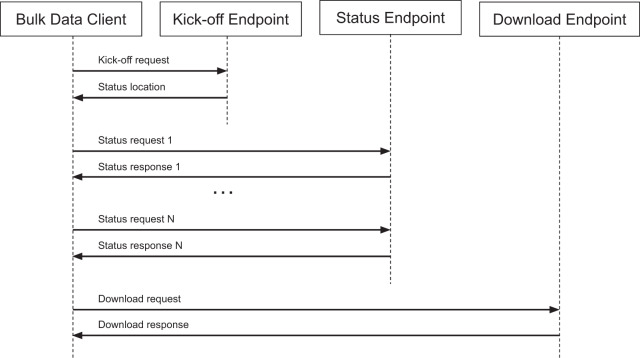
SMART/HL7 FHIR Bulk Data Workflow. The request workflow consists of a kickoff request, status requests to a generated status endpoint, and a file download request to hosted bulk data files.

Based on internet and FHIR standards^15,16^, the Bulk FHIR data flow following a kickoff request contains a kickoff response from the server, specifying a ‘Content-Location’ header with a location the client can poll for status requests. This location is then called in one or more sequential HTTP ‘GET’ status requests, culminating either in a ‘5XX’ error response or a ‘200 OK’ completion. Each status response can include a ‘X-Progress’ header, providing a text description of the server’s progress and/or a ‘Retry-After’ header to suggest the time a client should wait before sending the next status response. The completion response may contain an ‘Expires’ header with a timestamp until which it is able to host the prepared bulk files and must return a JSON manifest in its body with an array of bulk data file locations, potentially hosted on a separate file server. The last step of the data flow is an HTTP ‘GET’ file request to each of the file endpoints specified in the completion’s body. A successful full query flow is depicted in Fig. 1.

#### Authorization framework

**Fig. 2:**
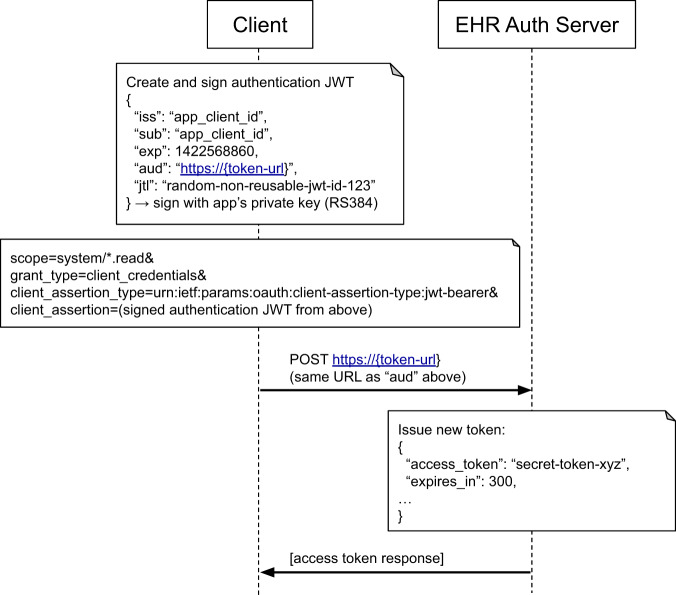
SMART Backend Service Authorization Workflow. An authentication JWT is posted to an EHR authorization server, which responds with an access token.

The specification advises servers to use SMART Backend Services Authorization to secure bulk data request. This authorization is designed to allow system-to-system communication with no additional human intervention after set up (see Fig. 2). In each session, the client creates a signed token request using its private key and a client id pre-assigned by the server. The server verifies this request with the client’s public key and issues a short-lived access token for use in the API requests. When the access token expires, the process is repeated. Because bulk data is not tied to the scope of a single patient, a client can use their identifier and key to request a broad “system-level” scope of the form “*system/[resourceType].read”* for the resources requested for reading. Servers may further limit the data return based on policies associated with the client’s account, for example, by restricting a payor organization to data on patients enrolled in one of the organization’s plans.

### Tools

To support bulk data implementations, the SMART Health IT team has developed a suite of open-source software artifacts.

**Fig. 3:**
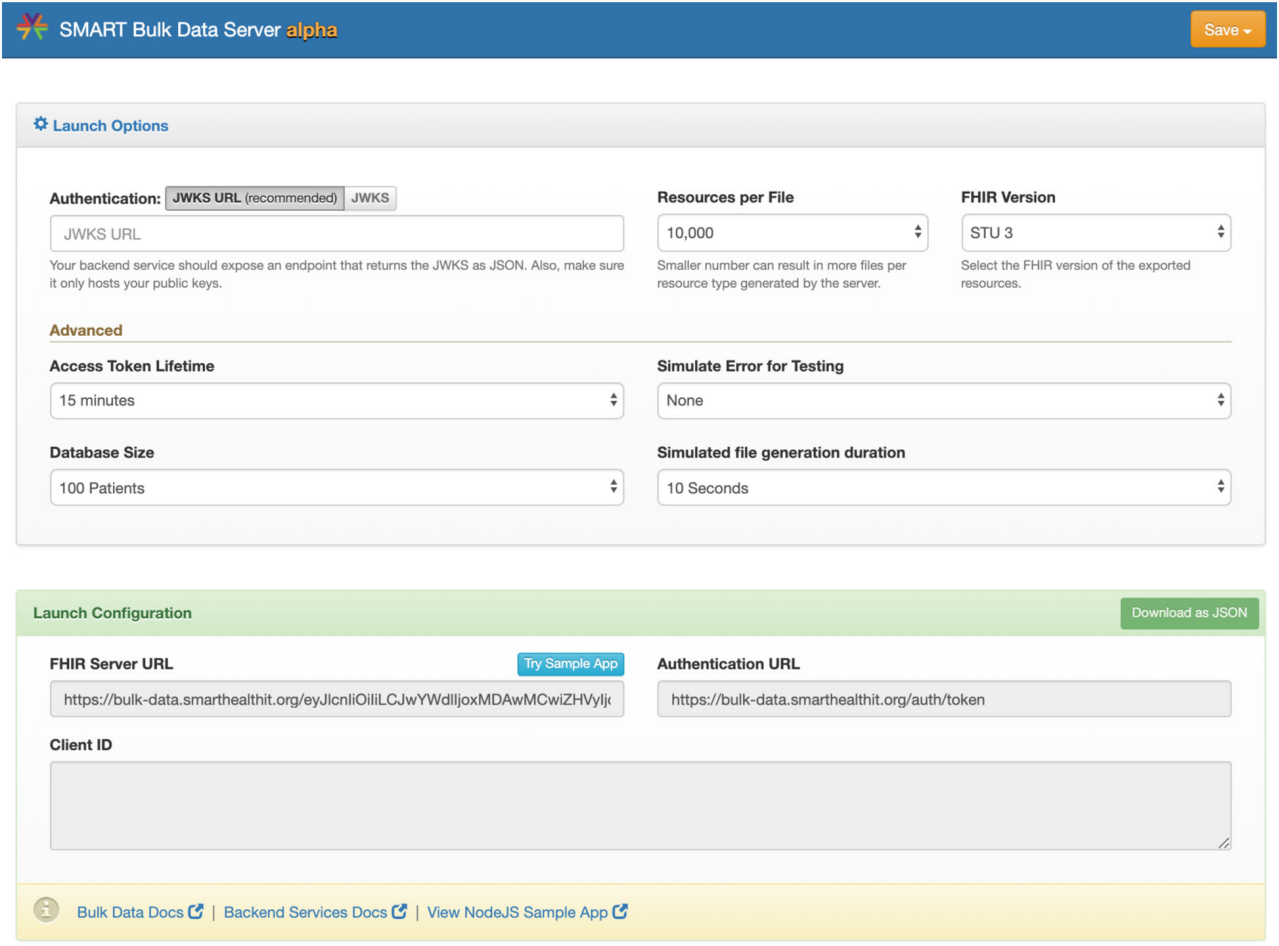
SMART Bulk Data Server Reference Implementation. The open-source SMART Bulk Data Server implements the Bulk Data Access API and serves synthetic data.

**Fig. 4:**
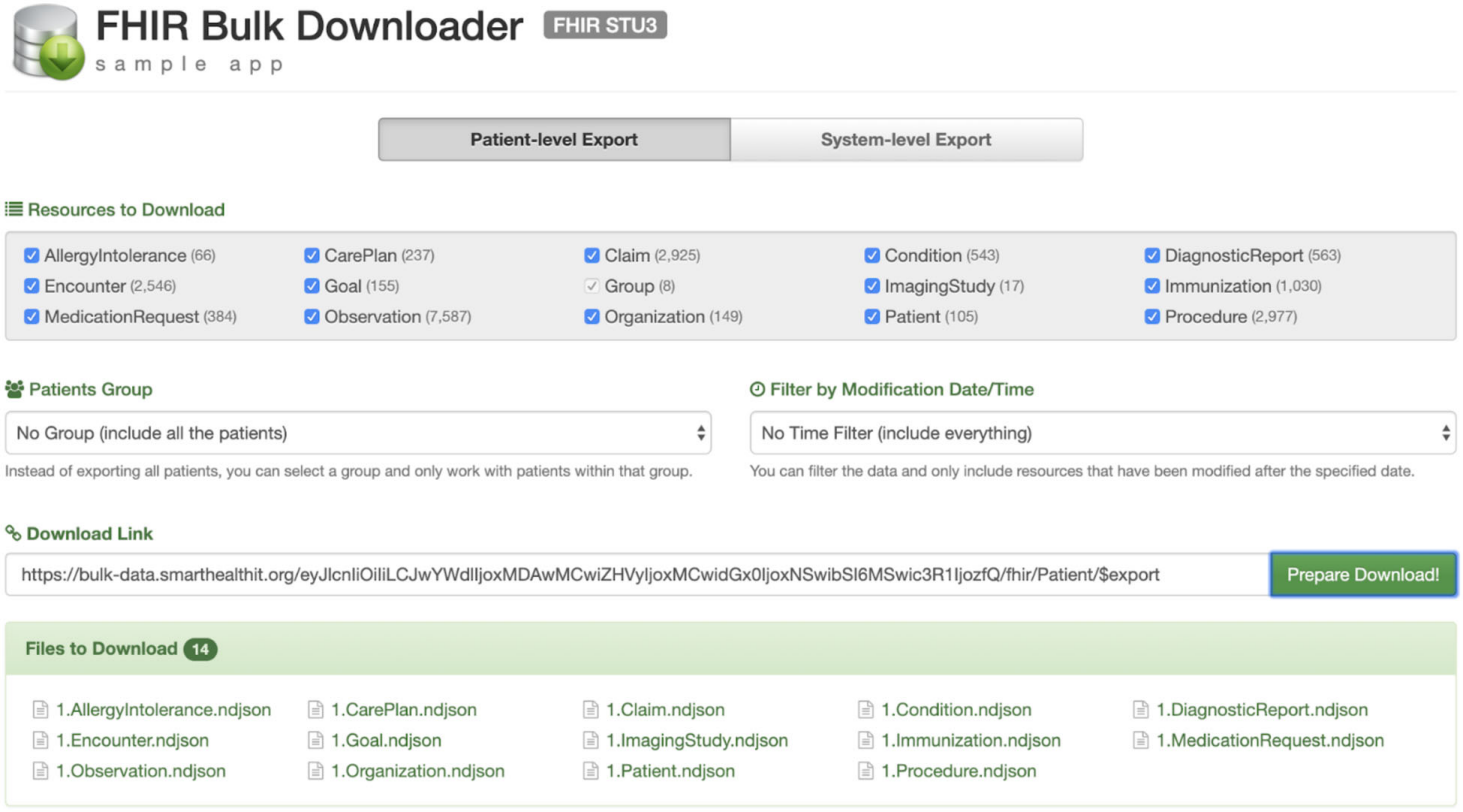
Bulk Data sample app. An open-source browser-based app provides an interface for downloading data from the SMART Bulk Data Server reference implementation.

The *Bulk Data Reference Server* (https://bulk-data.smarthealthit.org/) is designed for use by client software developers (Fig. 3). The reference server supports the SMART/HL7 FHIR Bulk Data Access Standard version 1.0 completely, except for the experimental “_typeFilter” parameter and returns synthetic FHIR data. It also supports many of the new features defined in version 1.5. To support testing multiple scenarios, parameters in the server user interface can be adjusted to simulate different behaviors, including injecting errors at different points in the bulk data request flow. The server includes a browser-based bulk data sample app designed to be launched from within the bulk data server configuration page (Fig. 4), useful for exploration and demonstrations. Code for the server is available at https://github.com/smart-on-fhir/bulk-data-server.

The *Bulk Data Client* (https://github.com/smart-on-fhir/sample-apps-stu3/tree/master/fhir-downloader) is a command-line client written in Node.js. The client includes a detailed graphical output and can be used to download bulk data as NDJSON from any standard-compliant bulk data server, with full support for SMART Backend Services Authorization.

**Fig. 5:**
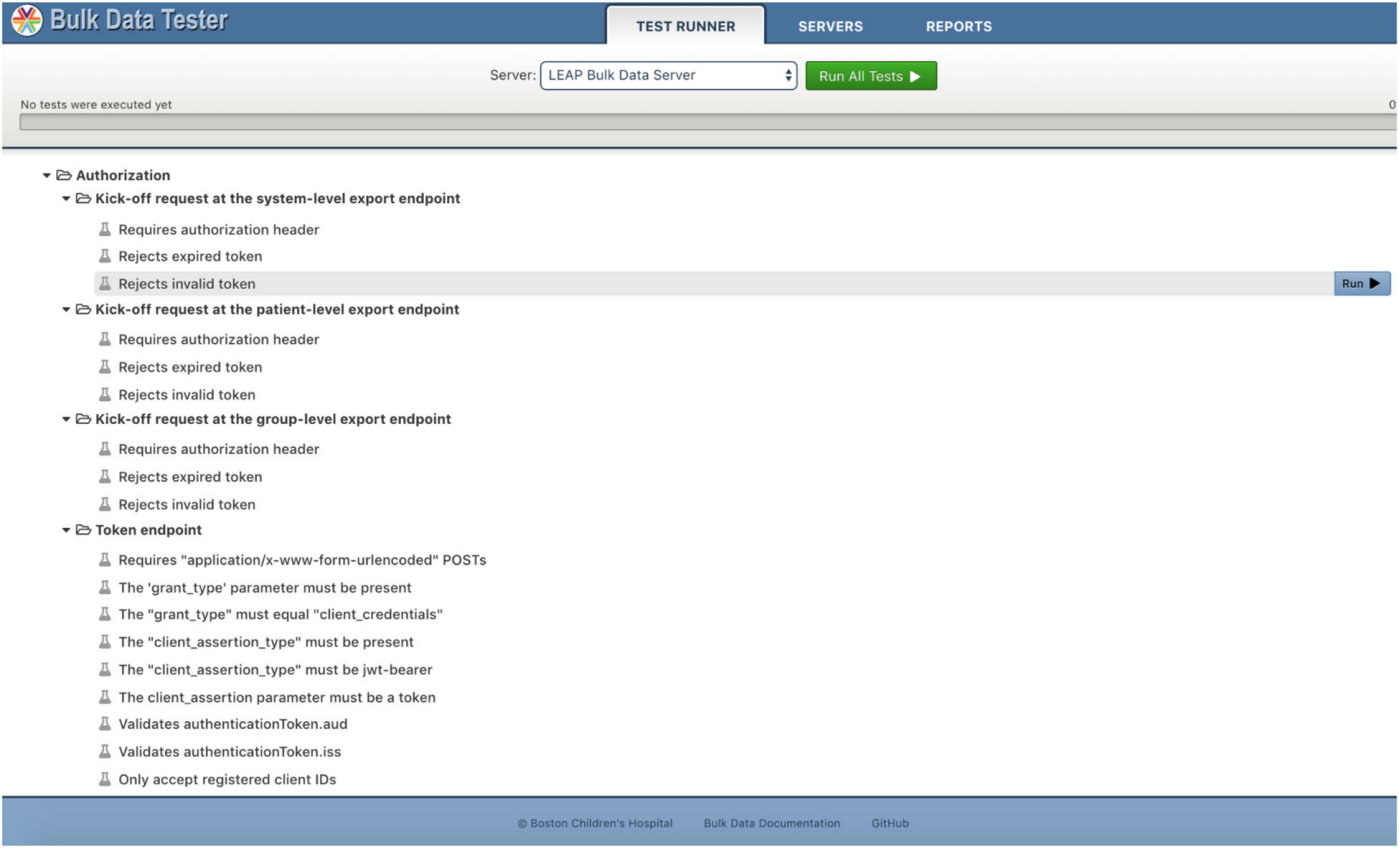
Bulk Data testing tool. The open-source Bulk Data Tester hosts a suite of tests to confirm Bulk Data Access API conformance.

The *Bulk Data Tester* (https://bulk-data-tester.smarthealthit.org/) is an open-source test suite (Fig. 5) and test runner for bulk data servers that can be used online or offline as a command-line interface or as a remote service using its public HTTP testing API.

**Fig. 6:**
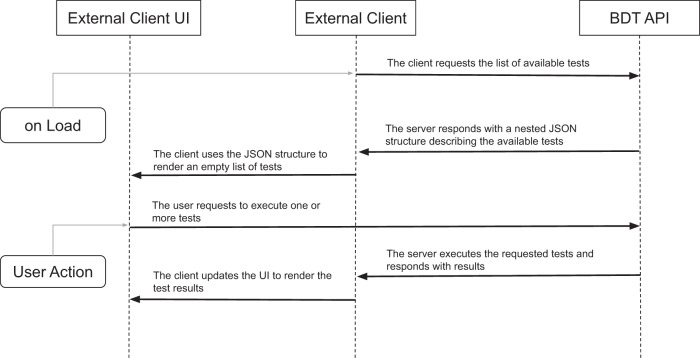
Substitutable FHIR Bulk Data testing. The open-source Bulk Data Tester API can be loaded through a web-based client for rendering available tests, allowing a customizable user interface.

The bulk data testing tool has three major components: (1) the test runner, where the actual test suite is located; (2) saved servers and configurations, useful for running repeated tests against a server; and (3) a report section allowing all users to view and compare published test servers. The SMART Bulk Data Tester can either be launched from the SMART website (https://bulk-data-tester.smarthealthit.org), or from the ONC’s Inferno FHIR testing tool (https://infernotest.healthit.gov/). The Bulk Data Tester API can be used from within a web app, as shown in Fig. 6. When the web app is loaded, it makes an initialization request to the Bulk Data Tester API server. The server responds with the list of available tests, including their names, descriptions, and other metadata and hierarchy information. The web app uses that to render its user interface. The app user can choose to run a single test, a group of tests, or all of the available tests. The web app sends that command to the back end and receives the result response when the tests are complete. The app can then update its interface to show which tests were successful and which failed, incorporating corresponding error messages where applicable.

### Standardization

The HL7 Bulk Data Implementation Guide was tested at a number of FHIR Connectathon events in 2018 and 2019, refined through a series of Argonaut Project working group meetings in 2018, and balloted through the HL7 process at the end of that year and into 2019, prior to being released as a HL7 STU1 standard on August 22, 2019. The current version of the IG is hosted at https://hl7.org/fhir/uv/bulkdata/index.html.

### Community uptake

Table 2 shows examples of substantial bulk data deployments. CMS is leveraging the standard for two major initiatives. *Data at the Point of Care* (https://dpc.cms.gov/) is a pilot project giving physicians access to patient claims data. Fee for service providers supply rosters of active patients and CMS returns FHIR-formatted bulk data files. The *Beneficiary Claims Data API* (https://bcda.cms.gov/), provides accountable care organizations that participate in a Shared Savings Program access to specific Medicare claims data, in bulk FHIR format, for their assignable/prospectively assigned beneficiaries. These two CMS programs ensure that provider organizations will have access to both EHR and claims data in compatible, computable formats, across modern interfaces. This combination enables both the depth and detail contained in EHR data—notes, laboratory results etc.—with the breadth of claims data—information from every site of care, sometimes referred to as a 360 view of the patient.

**Table 2:** Examples of existing SMART/HL7 Bulk FHIR export implementations.

Organization	Deployment	Website
CMS	Data at the Point of Care	https://dpc.cms.gov/
CMS	Beneficiary Claims Data API	https://bcda.cms.gov/
Google	Production cloud implementation planned	https://cloud.google.com/healthcare/docs/how-tos/fhir-import-export
Smile CDR	HAPI Server	https://hapifhir.io/
Microsoft	Microsoft FHIR Server	https://azure.microsoft.com/en-us/services/azure-api-for-fhir/
IBM	IBM FHIR Server	https://github.com/IBM/FHIR

Strong support for the Bulk Data API was demonstrated at the 2019 White House CMS Blue Button Developers Conference, when numerous groups pledged to test the bulk data API^17^. On the basis of these commitments for support, we anticipate that healthcare providers and payors, working with technology vendors, will make testing endpoints available over the next 12 months, allowing for feedback ahead of the 2022 regulatory deadline for support. During this same time period, the Argonaut Project has undertaken to consolidate feedback and propose API improvements in the form of a “v1.5” release of the Bulk Data IG. In addition, there is substantial community interest in defining a robust *$import* operation and standard analytic approaches along with associated reference implementations.

## Discussion

The original SMART on FHIR project took nearly 11 years from the initial proposal of an open and universal healthcare API^10^ to being instantiated in the ONC Final Rule^1^. In contrast, it was only a few months after the initial kickoff that CMS was piloting FHIR bulk data for provisioning claims to ACOs, and just over 2 years before the API was required under the Department of Health and Human Services regulations. There are a few possible reasons for the differential timelines. One is that moving large datasets around to address the measurement of value and pay for performance was important to influential stakeholders. Second, the efforts required to implement FHIR and SMART on FHIR across EHR products and in the CMS Blue Button Project (enabling individual access to beneficiary claims across a SMART on FHIR API), established a foundation from which to implement bulk FHIR for both EHR and claims data. Third, because of the success of FHIR and SMART on FHIR, there was a high level of confidence and enthusiasm in the policy and technology communities.

The ONC, HL7, and the SMART team continue to collaborate with the community to extend the SMART/ HL7 FHIR Bulk Data Standard so that it addresses additional use cases and is responsive to the needs of the healthcare community. Next steps include supporting and promoting implementations, cataloging community extensions to the standard, incorporating them into future versions, and updating the IG as the community gains implementation experience.

Streamlined access to bulk EHR data exports will underpin new efficiencies in exchanging data across populations. Sharing data inter-institutionally will occur, as always, within the bounds of contracts that specify exactly what data will be shared for what purpose, and under what obligations. However, with an efficient API, duly authorized users can soon be granted live access to bulk data on populations.

Because for both regulated APIs the data required to be exposed are limited to the USCDI—which will expand over time—data not in the core will need to be exchanged through other methods, or through custom local extensions to the specification.

The universal capacity system-to-system exchange of huge datasets will no doubt generate questions about privacy. The regulatory framework is no different for data accessed using the Bulk FHIR API than for any other data. A covered entity, as defined under the Health Insurance Portability and Accountability Act (HIPAA) will move its data under standard HIPAA requirements, with less burden on the organization to perform manual ETL processes. The data extracted from a provider organization are protected under the same privacy and security laws, and are only shared, for example, with a payor within the bounds of the existing contract, and vice-versa. The community is iterating upon methods to further address this issue, by standardizing additional experimental query parameters to request only specific data elements be returned.

We anticipate significant activity around developing the tooling and resources needed to continue building the FHIR Bulk Data ecosystem, including the creation of data pipeline components for common tasks such as de-identification, terminology transformation, data filtering, and natural language processing (NLP) of the free text in clinical notes.

There is substantial interest in bulk FHIR by federal agencies. FDA is testing its use for real-world evidence generation and the CDC is testing the API for use in biosurveillance. The National Institutes of Health (NIH)^18^ and Agency for Healthcare Quality and Research (AHRQ)^19^ have explicitly called for the use of SMART and FHIR APIs in research.

The law regulation should be viewed as a starting point. To be successful, the Bulk FHIR technologies must be widely tested and refined based on real-world experience. Fortunately, the ONC regulation neither ties standardization to one set of data types—the USCDI will expand—nor to a static implementation guide. The regulation allows for iteration, learning, and future balloting of improvements.

Importantly, use of the API must be affordable. There are computing costs associated with data exchange and manipulation. The decisions of who bears the burden for these costs, and how much additional cost a provider should incur to retrieve data they have already entered into an EHR they have already purchased, will be key business drivers determining the level of data liquidity and therefore, the benefits accruing to the health-system writ large.

## Methods

### Approach

The SMART/HL7 FHIR Bulk Data Access API was designed primarily to address the cumbersome manual process of extracting data from EHRs. SMART on FHIR API technologies^20,21^ were leveraged as a starting point toward efficient population-level queries. The Bulk Data API expands the base SMART on FHIR API to address key desiderata around system-level export operations across the full spectrum of the FHIR data model. First of all, the Bulk Data API implements a standardized asynchronous request pattern that supports data streaming, producing NDJSON flat files (“flat FHIR”) for efficient use in analytic engines. The asynchronous pattern allows servers and clients to more gracefully handle large requests, as some of these exports may take considerable memory to prepare and process. For computational efficiency post-export, the flat FHIR formats recognize the diverse structures of FHIR resources and store them in a compressible format with one data type per file.

All other decisions for the Bulk Data API facilitated reuse of existing FHIR semantics wherever possible, including the design of an automated back-end validation that builds upon the widely adopted OAuth standard used in SMART on FHIR—specifically the SMART Backend Services Authentication and Authorization as a security model.

### Building the ecosystem

In order for the SMART/HL7 FHIR Bulk Data Access API to be used broadly and effectively to advance the use cases described, we placed strong emphasis on fostering a robust ecosystem of developers and end-users ready to support this work. Organizations including HL7 have coordinated numerous presentations and Connectathon events (Table 3) within the United States and abroad to support the advancement and development of the FHIR standard. The SMART Health IT team attended and participated in many of these events, leading FHIR Connectathon tracks and regularly presenting on the Bulk Data API to inform and engage with the community on its uses, current support, future goals, and further, to test the technology across community use cases. In November of 2019, HL7 and the SMART Team hosted, on behalf of ONC, a second Bulk FHIR Export meeting, where there was enthusiastic support for use cases across federal agencies, including CMS, National Institutes of Health, CDC, and the Food and Drug Administration (FDA)^22^.

**Table 3:** SMART/HL7 FHIR Bulk Data Access API Meeting for development and promotion.

Event	Location	Date
EHR Population Level Data Exports to Support Population Health and Value Meeting	Boston, MA	December 2017
HL7 FHIR Connectathon Bulk Data Track	New Orleans, LA	January 2018
HL7 FHIR Connectathon Bulk Data Track	Cologne, Germany	May 2018
HL7 FHIR Connectathon Bulk Data Track	Baltimore, MD	September 2018
HL7 FHIR Connectathon Bulk Data Track	San Antonio, TX	January 2019
HIMSS Global Health Conference & Exhibition Presentation	Orlando, FL	February 2019
HL7 FHIR Connectathon Bulk Data Track	Montreal, Canada	May 2019
HL7 FHIR DevDays US Presentation	Redmond, WA	June 2019
HL7 FHIR Connectathon Bulk Data Track	Atlanta, GA	September 2019
Meeting to Advance Push Button Population Health: SMART/HL7 Bulk Data Export/FLAT FHIR	Boston, MA	November 2019
CMS FHIR Connectathon Bulk Data Track	Baltimore, MD	January 2020
ONC Annual Meeting Presentation	Washington, DC	January 2020
HL7 Connectathon Bulk Data Track	Virtual	May 2020
HL7 FHIR DevDays Presentation	Virtual	June 2020

### Argonaut

Prior to balloting and publication, the initial version of the Bulk Data IG was refined in collaboration with implementers through the Argonaut FHIR Accelerator working group process in 2018. In 2020, a new Argonaut workgroup has been launched to incorporate learnings from pilots and early implementations a future version of the specification. Topics being addressed include handling binary content in exports (such as PDF documents associated with a patient), improvements to incremental update handling (such as receiving historical data for new members of a group and propagating resource deletions to client applications), documentation clarifications (such as parameter optionality for servers and the use of POST requests), handling metadata about the resources being returned (such as provenance resources), and export job management improvements (such as cleaner error guidance and the ability for clients to signal the server when files may be removed).

### Balloting

In the Spring 2019 HL7 ballot cycle, 177 participants signed up to review the Bulk Data 1.0 Implementation Guide, contributing 116 questions, comments, and suggestions. The HL7 FHIR Infrastructure Workgroup adjudicated these ballot comments, leading to improvements in the clarity of the specification, and additional features for discovering server capabilities, refining the exported dataset, and managing the export job lifecycle. The resulting “Standard for Trial Use” document was published in August 2019.

### Regulatory approach

In the spring of 2019, ONC proposed a rule to implement the 21st Century Cures Act API and information blocking provisions^1^. While Bulk FHIR Access was not an explicit requirement of the Proposed Rule, ONC integrated this specification by name into the Final Rule^1^, based on substantial progress in the community, and real-world implementations, e.g., by CMS.

### Reporting summary

Further information on research design is available in the Nature Research Reporting Summary linked to this article.

## Supplementary information

Reporting Summary Checklist

## Data Availability

No human data were used in this study. All synthetic sandbox data are publicly available at the URLs cited.

## Code availability

All software and specifications are publicly available at the URLs cited.
